# Diet-driven interferon-*γ* enhances malignant transformation of primary bovine mammary epithelial cells through nutrient sensor GCN2-activated autophagy

**DOI:** 10.1038/cddis.2016.48

**Published:** 2016-03-10

**Authors:** X-j Xia, Y-y Che, J Zhang, Y-y Gao, C-j Ao, H-j Yang, J-x Liu, G-w Liu, W-y Han, Y-p Wang, J-q Wang, L-C Lei

**Affiliations:** 1 College of Veterinary Medicine, Jilin University, Changchun, China; 2 Affiliated Hospital's Preparation Center, Changchun University of Chinese Medicine, Changchun, China; 3 College of Animal Science, Jilin University, Changchun, China; 4 College of Animal Science, Inner Mongolian Agricultural University, Hohhot, China; 5 College of Animal Science and Technology, China Agricultural University, Beijing, China; 6 Institute of Animal Science, Chinese Academy of Agricultural Science, Beijing, China

Cytokines are related to breast cancer, which has one of the highest incidence rates of cancer in women worldwide. Breast cancer has been gaining intense attention;^1^ however, various uncertain ‘hidden risk factors' for cytokines in the promotion of breast cancer have been reported, such as long-term exposure to diets that increased the levels of certain inflammatory factors. Interferon gamma (IFN-*γ*) has been used successfully to treat various solid cancers and haematological malignancies, but limited and transient effects have been noted in breast cancer patients. A steady flow of reports suggests that IFN-*γ* may also have pro-tumorigenic effects under certain circumstances and that IFN-*γ* may even enhance cancer progression.^2^ What would happen in the breast if a body maintains high levels of IFN-*γ* for long periods of time because of special diets or food preferences? We identified a cow model to indicate that straw diet-driven IFN-*γ* could enhance autophagic activity in mammary epithelial cells *in vivo*. To our surprise, we found that autophagy, which is mediated by arginine depletion and activation of the nutrient sensor GCN2 due to IFN-*γ* treatment, promotes the IFN-*γ*-induced malignant transformation in primary bovine mammary epithelial cells (BMECs) *in vitro* (Figure 1).^3^ These findings strongly provide an improved understanding of the relationship between diet and breast cancer through the bridge of cytokines, and suggest the rational use of IFN-*γ* in the clinic. These results also provide new directions and paths for preventing and treating breast cancer via diet.

Exploring the dangerous factors that cause cancer is the key to reducing the rates of cancer. Currently, established risk factors for breast cancer have been identified, including family history, hormone therapy, age, and obesity. One of the most modifiable risk factors is diet.^4^ In the development and evolution of cancer, the repair of anti-cancerous cells depends on factors received from the surrounding microenvironment, including the use of energy and a moderate amount of nutrients.^5^ However, these existing reports focused on detecting IFN-*γ* in cancer patients. Direct experimental evidence that suggests whether diet directly stimulates the production of IFN-*γ* and potential hazards in the healthy body is not available. Here we describe the process whereby a corn straw diet increases IFN-*γ* levels in cow milk and serum, which subsequently induces autophagy in BMECs *in vivo*. In addition, constant exposure to IFN-*γ* results in malignant transformation of BMECs *in vitro*.^3^ As early as the 1860s, Virchow recognised the relationship between inflammation and tumours, and first described the existence of white blood cell infiltration in tumour tissues. Virchow indicated that tumours often occurred at the site of chronic inflammation.^6^ In an inflammatory microenvironment, inflammatory cells release a large number of reactive oxygen species and reactive nitrogen mediators, induce DNA damage and genomic instability, and increase the frequency of gene mutations.^7^ The instability and mutation of oncogenes increase genetic diversity, which is beneficial to form a heterologous genome that possesses increased selectivity for proliferation, invades anatomically distant normal tissues, escapes from the immune defence mechanism of the host, and initiates tumour. These results may provide insightful information for human clinical trials.

Clinically, IFN-*γ* is therapeutically used for bladder carcinoma, ovarian cancer, and breast cancer.^8^ However, the results have been mixed (reviewed in Miller *et al.*^9^). In the clinic, only a limited number of patients are sensitive to IFN-*γ* treatment, suggesting that the function of IFN-*γ* in the development and progression of cancer is complicated. Our recent study reported that long-term constant exposure to IFN-*γ* results in malignant transformation of BMECs *in vitro*.^3^ To date, the IFN-*γ* treatment duration has not been optimised as this agent is more beneficial for inducing remission and improving survival in oncologic applications. Therefore, specific interventions to achieve maximum therapeutic effect should be developed.

Autophagy is a double-edged sword in breast cancer. Autophagy can act as a cancer suppressor. However, an increasing number of studies have demonstrated that autophagy may be involved in the initiation and/or promotion of cancer.^10^ We demonstrate that IFN-*γ* induces malignant transformation via autophagy, which is mediated by arginine depletion and activation of GCN2 in primary BMECs *in vitro*.^3^ Guo *et al.*^11^ reported that autophagy promotes tumour growth by suppressing the p53 response, maintaining mitochondrial function, sustaining metabolic homoeostasis and survival under stress, and preventing the diversion of tumour progression to benign oncocytomas. As a corollary, autophagy may promote tumour development, indicating that autophagy inhibition might be a requirement for inhibiting tumorigenesis and may serve as a valid approach for cancer therapies.

In experimental settings, the pharmacological inhibition of autophagy is often realized with the administration of hydroxychloroquine (HCQ), a lysosomotropic drug approved by the US Food and Drug Administration. Current clinical trials indicate that HCQ can be safely employed alone or in combination with other drugs to inhibit autophagy during breast cancer therapy.^12^ Intriguingly, we first identified that IFN-*γ* promotes arginine depletion, which is related to the activation of autophagy via GCN2 and involved in malignant transformation.^3^ GCN2 is a serine/threonine kinase that functions to detect a paucity of one or more essential amino acids and to send a signal that enables an appropriate adaptive response.^13^ Upon cancer cell activation, GCN2 can initiate autophagy to gain more energy for further growth. Currently, the role of GCN2 in breast cancer is poorly understood. In the future, research should be performed to understand its characteristics and aid in the design of drugs or diagnostic molecules targeted to breast cancer.

Diet is modestly associated with the breast cancer risk. Pro-inflammatory cytokine levels may be associated with the cancer stage, recurrence, and survival. An increased consumption of plant-based foods was associated with a 15% reduction in breast cancer risk.^14^ Our previous findings confirm that a corn straw diet alters IFN-*γ* levels in healthy cows, and diet-driven IFN-*γ* enhances malignant transformation of primary BMECs through nutrient sensor GCN2-activated autophagy.^3^ Taken together, the antitumour effect of IFN-*γ* remains a problem to be conquered. We first described the strong correlations among nutrition, immunometabolism, autophagy, and cancer, suggesting that diet might be an effective mechanism for preventing breast cancer. This information may provide new insight into breast cancer prevention and therapy.

## Figures and Tables

**Figure 1:**
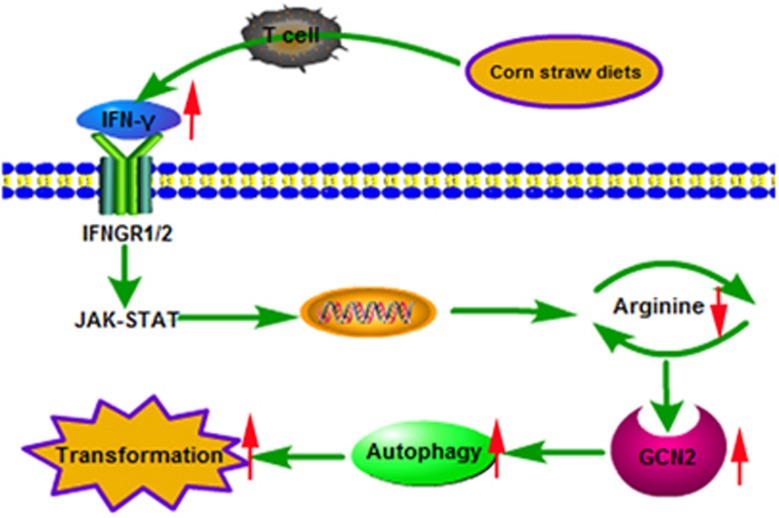
The model of IFN-*γ*-induced autophagy in BMECs in dairy cow mammary glands. The depletion of arginine leads to the activation of GCN2 kinase-mediated IFN-*γ*-induced autophagy. This constant activation of autophagy promotes BMEC migration and invasion *in vitro* and *in vivo*
